# Is weight just a number? The accuracy of UK ambulance paediatric weight guidance – findings from a cross-sectional study

**DOI:** 10.29045/14784726.2020.12.5.3.1

**Published:** 2020-12-01

**Authors:** Karl Charlton, Matt Capsey, Chris Moat

**Affiliations:** North East Ambulance Service NHS Foundation Trust; Teesside University; Teesside University

**Keywords:** body weight, emergency care, emergency medical services, paediatrics, pre-hospital care

## Abstract

**Background:**

The weight of children provides the cornerstone of their clinical management, as many drug dosages, equipment sizes, fluid boluses, as well as DC shock energy, are administered on a per kilogram basis. Children who attend hospital are weighed using scales prior to receiving these interventions. This is not possible in the pre-hospital environment. A paucity of evidence exists to support the page for age weight guidance indicated by JRCALC, and it remains unknown if this approach meets the reference standard of 70% of estimations within 10% of actual weight and 95% within 20% of actual weight.

**Methods:**

We used a cross-sectional study design and collected data from a convenience sample of children who attended the outpatients department of a major hospital in England between July and September 2019. All children aged between 1 and 11 years who were weighed were eligible for inclusion. Outcomes were to determine if the page for age approach meets the reference standard and to determine any implications for care.

**Results:**

341 children were included in this study. Each age group consisted of varying numbers of children. 50.5% (172/341) of the sample were female. Observed weights ranged from 8.28 to 82.70 kg (median 20.60 kg). The mean weight of girls versus boys was 24.69 kg and 23.39 kg respectively (95% CI -4.12–1.32, p = 0.3123). Observed weights were greater than the page for age guidance weight in all age groups, and the accuracy of page for age weight guidance diminished with age. Adrenaline 1:10,000 doses and defibrillation energy levels guided by page for age differ from those guided by weight, but are not deleterious to care.

**Conclusion:**

Page for age weight guidance does not meet the reference standard. Most paediatric pre-hospital care is administered by age and not weight. In the absence of an accurate weight, ambulance clinicians should continue to use the page for age system, although the gold standard remains to use an accurate weight measurement. While there are no facilities to weigh children in ambulances, if an accurate weight is available then consideration should be given to using this rather than age.

## Background

In paediatric paramedic medicine, it is important to know a child’s weight in order to calculate therapeutic interventions. The weight of children provides the cornerstone of their clinical management, as many drug dosages are calculated on a per kilogram basis, as are equipment sizing, fluid boluses and DC shock energy (Luscombe et al., 2010). The ‘gold standard’ method of determining a child’s weight is to weigh them using scales (Argall et al., 2003). The practicalities of carrying scales in an ambulance and the nature of the emergency situation encountered by paramedics preclude the use of such methods. However, the requirement for a convenient, quick and accurate method of calculating weight is no less important.

The United Kingdom (UK) ambulance service clinical guidelines (JRCALC) offer a suggested page for age mean weight for all children aged between 1 and 11 years of age (JRCALC, 2019). For children aged between 1 and 4 years of age, weight is calculated using the advanced paediatric life support (APLS) (Advanced Life Support Group, 2005) formula of (age x 2) + 8. Weight guidance for children aged 5 to 7 years of age uses the formula (age x 2) + 9, and for children aged 8 to 11 years of age the formula is (age x 3) + 2. The age is the child’s age in whole years at their last birthday. Inaccurate weight guidance may lead to inappropriate or inadequate treatments, leading to potential suboptimal health outcomes. A paucity of evidence exists to support the use of current UK ambulance weight guidance, which may contribute towards the provision of poor out-of-hospital care; consequently, a knowledge deficit exists in current paramedic practice. We set out to frame the evidence base for the weight guidance currently in use.

## Objectives

Research suggests that any weight estimation formula should achieve a minimum accuracy of 70% of estimations within 10% of actual weight and 95% within 20% of actual weight before being considered accurate, and that this should be considered the reference standard (Wells et al., 2017). We set out to determine if current page for age weight guidance achieves these accuracy thresholds and to assess the impact of any differences. Our starting hypothesis accepted equivalence between page for age mean weight and the observed weight of children when weighed in hospital.

Findings are reported in accordance with the strengthening the reporting of observational studies in epidemiology (STROBE) statement (Von Elm et al., 2007).

## Methods

Data were recorded for a convenience sample of children aged 1 to 11 years who attended the outpatients department of James Cook University Hospital, Middlesbrough between July and September 2019 inclusive. Nursing staff collected the age in whole years and the sex and weight in kilograms (to two decimal places) of each child. Children were weighed using Seca 888, Seca 861 and Seca 727 scales, which were regularly calibrated in accordance with manufacturers’ recommendations. Weights were recorded with all children wearing minimal, light clothing and without coats and footwear, as this is standard practice (Child Growth Foundation, 2012).

Anonymised study data for each child were forwarded by the research team at James Cook University Hospital to the researcher at North East Ambulance Service NHS Foundation Trust via secure NHS email.

## Data analysis

Data were analysed within each age group to facilitate comparative analysis. With support from a statistician, data were analysed using MedCalc version 19.0.5. All data were normally distributed. Differences between observed weights between sexes were evaluated by a repeated measures t-test. Differences between observed and guidance weights for each age group were evaluated using a X^2^ test and are reported with 1 degree of freedom (*df*). For all cases, a p value of < 0.05 was considered statistically significant.

## Results

**Figure 1:**
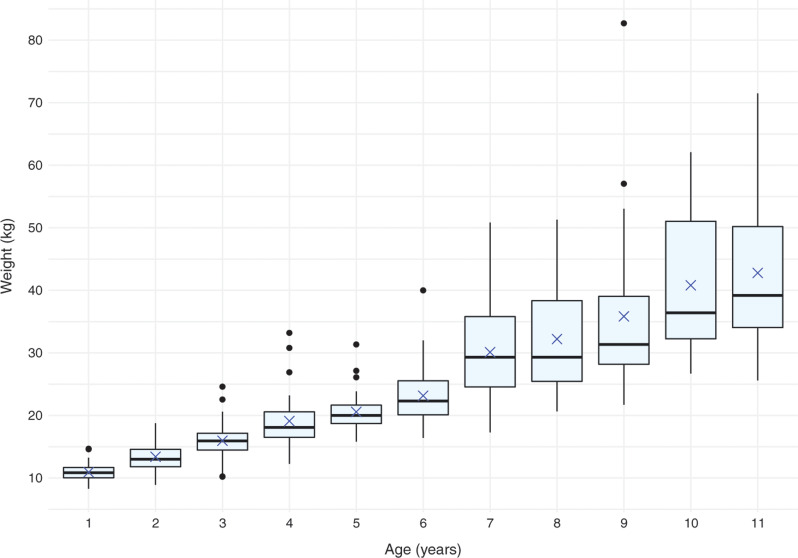
Figure 1. Observed weight distribution by age (middle line denotes median observed weight; x denotes mean observed weight).

All eligible children who attended the outpatients department between July and September 2019 inclusive were included, resulting in data from 341 children being available for analysis. All data sets analysed involved children aged between 1 and 11 years inclusive, and no children had missing data. Each age group consisted of varying numbers of children. Observed weights ranged from 8.28 to 82.70 kg (median 20.60 kg). The observed weights of children in the older age groups (7–11 years) were more widely distributed than those in the younger age groups (1–6 years) (Figure 1).

The total sample was balanced between both sexes (50.5%, 172/341 female) but both sexes were represented differently in each age group. The mean weight of all the girls and boys in our sample was 24.69 kg and 23.39 kg respectively (mean difference 1.3 kg, 95% CI −4.12–1.32, p = 0.31). In some groups (6/11), girls outweighed boys, but the differences between the observed weights of both sexes did not achieve statistical significance in any age group (Table 1).

**Table 1.** Observed weight distribution by sex.

Age (yrs)	Female (n)	Male (n)	Mean female weight (kg)	Mean male weight (kg)	Mean difference (kg)	Mean difference 95% CI	p
**1**	22	19	10.6	11.3	0.70	−0.17–1.57	0.21
**2**	22	26	13.12	13.61	0.49	−0.88–1.86	0.47
**3**	11	17	16.02	15.91	0.11	−2.84–2.62	0.93
**4**	19	15	18.47	19.9	1.43	−1.63–4.49	0.34
**5**	15	15	20.98	20.15	0.83	−3.37–1.71	0.50
**6**	13	19	24.57	22.18	2.39	−5.92–1.14	0.17
**7**	11	17	27.38	31.8	4.42	−1.43–10.27	0.13
**8**	21	9	34.05	31.5	2.55	−11.06–5.96	0.54
**9**	14	10	36.08	34.48	1.6	−12.97–9.77	0.77
**10**	9	8	39.63	42.12	2.49	−10.03–15.01	0.67
**11**	15	14	44.98	40.44	4.54	−14.23–5.15	0.34
**Total**	172	169					
**Total mean weight**			24.69	23.39	1.3	−4.12–1.32	0.31

The observed weights in our sample were greater than the page for age guidance weight in all age groups. Page for age was more accurate for younger age groups, where differences between observed weights still existed but were not statistically significant. Differences in weight became more evident and statistically significant in older children (Table 2).

**Table 2.** Accuracy of JRCALC versus observed weight.

Age	*N*	Page for age	Observed weight	% accuracy	X^2^	p
**1**	41	10	10.93	91.49	1.91	> 0.05
**2**	48	12	13.38	89.68	3.57	> 0.05
**3**	28	14	15.96	87.71	3.47	> 0.05
**4**	34	16	19.1	83.76	9.25	< 0.01
**5**	30	19	20.56	92.41	1.86	> 0.05
**6**	32	21	23.15	90.71	3.27	> 0.05
**7**	28	23	30.11	76.38	26.65	< 0.001
**8**	30	26	33.31	78.05	26.67	< 0.001
**9**	24	29	35.83	80.93	17.28	< 0.001
**10**	17	32	40.81	78.41	17.96	< 0.001
**11**	29	35	42.79	81.79	22.46	< 0.001
**Total**	341					

Page for age guide weights did not achieve the reference standard of 70% of estimations within 10%, or 95% within 20%, of the observed weights (Table 3). Page for age guidance accurately determined the exact weight of a child in our sample only 1.17% of the time (4/341). The guidance weights consistently miscalculated observed weights; some weights were overestimated (24.63%) and others underestimated (74.11%), but page for age performed poorly throughout.

**Table 3.** Accuracy of ambulance weight guidance.

Age (yrs)	Page for age (kg)	Within 10% (n)	Within 20% (n)	More than JRCALC (n)	Less than JRCALC (n)	Exact (n)
**1**	10	23 (56%)	34 (82%)	32 (78%)	8 (20%)	1 (2%)
**2**	12	21 (44%)	33 (69%)	33 (69%)	14 (29%)	1 (2%)
**3**	14	7 (25%)	16 (57%)	23 (82%)	5 (18%)	0
**4**	16	13 (38%)	22 (65%)	30 (88%)	4 (12%)	0
**5**	19	4 (13%)	11 (37%)	25 (83%)	5 (17%)	0
**6**	21	15 (47%)	22 (69%)	19 (59%)	13 (41%)	0
**7**	23	7 (25%)	11 (39%)	23 (82%)	4 (14%)	1 (4%)
**8**	26	14 (47%)	16 (53%)	20 (67%)	10 (33%)	0
**9**	29	10 (42%)	12 (50%)	16 (67%)	7 (29%)	1 (4%)
**10**	32	5 (29%)	11 (65%)	13 (76%)	4 (24%)	0
**11**	35	8 (28%)	15 (52%)	19 (66%)	10 (34%)	0

While most medicines in the UK ambulance guidelines medicines formulary are not administered by or affected by weight, differences were identified between the dose of adrenaline 1:10,000 (10 micrograms per kg) and defibrillation energy (4 joules per kg) when compared to Paediatric Advanced Life Support guidelines (Resuscitation Council, 2015); see Table 4.

**Table 4.** Adrenaline 1:10,000 dosages and joules by page for age vs. observed weight.

Age (yrs)	Adrenaline 1:10,000 (10 micrograms per kg)		Joules (4j per kg)	
	Page for age	Observed	Page for age	Observed
**1**	100	110	40	44
**2**	120	134	50	52
**3**	140	160	60	64
**4**	160	191	70	76
**5**	190	205	80	84
**6**	210	231	80	92
**7**	230	301	100	120
**8**	260	333	100	132
**9**	300	358	120	144
**10**	320	408	130	164
**11**	350	427	140	172

## Discussion

There is currently a paucity of research evaluating the accuracy of UK ambulance paediatric weight guidance. Our study set out to evaluate if page for age, currently used in UK paramedic practice, is accurate, and to determine any impact upon clinical care. To our knowledge, this is the first study to explore this area of paramedic practice.

Page for age matched the observed weight of children in our sample only 1.17% (*n* = 4/341) of the time, and consistently underestimated weight in all age groups; the differences between the observed weights and guidance weights were clinically and statistically significant. The APLS weight formula (age x 2) + 8 used in ambulance guidance for children aged 1–4 years tended to be the most accurate, although inaccurate estimations remained. Our research is supported by others who question the validity and accuracy of this formula (Black et al., 2002; Marlow et al., 2011). Our findings also indicate that the accuracy of page for age diminishes with age, as the formulae used to underpin weight guidance for children aged 5–11 years were less accurate, and to date remains unvalidated. Our study comprised a broad range of weights in each age group, including significant outliers, and this raises questions about guidelines that suggest one weight per age.

Our main finding is that current page for age paediatric weight guidance does not accurately reflect the weight of children in our sample. However, further questions arise as to the clinical significance of this finding and whether it is deleterious to care. All drugs in the page for age guidance are given by age and are thus unaffected by inaccuracies in weight. This is not concordant with other medicines formularies, such as the British National Formulary (BNF) which recommends medicines are given to children by weight.

We explored how these differences impacted upon resuscitation management, and we focused on the doses of adrenaline 1:10,000 and defibrillation energy level as indicated by observed weight versus page for age. Accurate weight estimation is of paramount importance in paediatric resuscitation (Luscombe et al., 2010), and our research suggests that current page for age weight guidance may result in children receiving lower doses of adrenaline 1:10,000 and defibrillation energy level than would be suggested by their observed weight. However, evidence suggests that this may be advantageous, rather than deleterious, to favourable outcomes. In their study involving 68 children suffering an in-hospital cardiac arrest, Beatriz et al. (2004) assigned patients to receive either standard or higher rescue doses of adrenaline after their initial standard dose. Those receiving higher rescue doses of adrenaline had a lower rate of survival to 24 hours, as well as lower rates of survival to discharge. This is supported by retrospective studies that suggest that higher doses of adrenaline in paediatric cardiopulmonary arrest are not beneficial to outcomes and may actually be harmful (Carpenter & Stenmark, 1997; Dieckmann & Vardis, 1995).

Children present in ventricular fibrillation (VF) or pulseless ventricular tachycardia (VT) in approximately 3.8–19% of cardiopulmonary arrests, and the prevalence of shockable rhythms increases with age (Bray et al., 2014). However, the ideal energy level for safe and effective defibrillation in children is unknown (Ali & Bingham, 2018).

Simulations using animal models indicate better results with defibrillation energy levels of 3–4 joules per kg than adult energy levels (Berg et al., 2005), and there is no data to support any change in current practice. Furthermore, the devices commonly in use within UK ambulance services (Likepak 15 and Zoll X Series) deliver incremental defibrillation energy levels and cannot currently deliver the specific doses indicated by page for age or our findings. It appears that current doses of adrenaline and defibrillation energy levels as suggested by page for age are safe and within therapeutic ranges.

There are advantages to age-based weight formulae that make them particularly applicable to paramedic practice. They are simple and quick to use. They require no equipment and, provided the correct age is known, can allow a paramedic to begin to calculate medicine doses en route to the location of the emergency. In addition, age-based formulae lessen the risk of human error when calculating emergency medicine doses, in situations of heightened pressure and in a healthcare population who rarely require these skills in clinical practice. However, the exact age of a child is not always known by the attending paramedic and several studies show that age-based formula underestimate weight in first world populations and are variable by age and ethnicity (Britnell & Koziol-McLain, 2015; Cattermole et al., 2011). In addition, current JRCALC guidance allows for clinician perceptions to influence care and suggests that when a child appears to be over or under the expected weight for their age, the previous or next page for age can be used (JRCALC, 2019).

Our findings question both the confidence with which a weight can be estimated based on age and the weights upon which current page for age guidance is based.

Several alternative weight estimation methods exist. In a recent systematic review by Wells et al. (2017), length-based weight estimations were found to consistently outperform age-based weight estimations. This suggests that using length to determine weight is biologically valid (Goldman et al., 2015).

However, none of the length-based estimates met the reference standard, and the practicalities and necessity of using a length-based formula in paramedic practice remain unexplored. In their study, Krieser et al. (2007) collected data from 410 children who attended ED and found parental estimations to be 78% accurate to within 10% of measured weight. Although this method exceeds the reference standard, potential limitations to its use remain. Some parents may be unwilling to disclose their child’s weight or, if the child has not been weighed recently, may be unable to do so with any degree of precision. It is also suggested that mothers may have more knowledge of their child’s weight than other caregivers (Krieser et al., 2007), so the utility of this method may rely on who is present with the child. Furthermore, the ability of a parent to accurately recall their child’s weight may be impaired when the child is critically unwell. We did not compare length-based or parental weight estimations to the page for age method, and so cannot make any conclusions as to how they compare.

All weight estimation formulae have limitations and result in a degree of imprecision. What degree of over or under estimation of weight is harmful to children remains unclear (Thompson et al., 2007).

Page for age weight guides, and the drug doses that derive from them, remain a quick and easy-to-use system. When an accurate weight is available, consideration should be given to using this rather than an age-based guide. Care derived from an accurate weight remains the gold standard, and page for age is not a substitute for safe, clinical acumen. Where no weight is available, page for age remains the best/only option and clinicians should use it in addition to their clinical judgement. As paramedics move into prescribing roles, use of page for age will be insufficient to underpin practice and a more accurate medicines formulary supported by weight will be required.

## Strengths and limitations

Our data were prospectively collected within the last 12 months, meaning they are current and are an accurate reflection of the weight of children in our sample.

Our data are limited in that we did not collect additional demographic variables such as ethnicity, which may impact upon the generalisability of our findings. Our data were limited to one hospital in the North East of England, an area known to suffer higher than average childhood obesity (NHS, 2018), and this may account for the children in our sample exhibiting weights heavier than the national guidance. Furthermore, we collected our observed weights from the paediatric outpatients department. While we believe the children in our sample to be representative, they may differ in general health, and consequently weight, from the wider paediatric population.

We were unable to collect data from a similar number of children in each age group, as we were restricted by the children who attended the outpatients department throughout the recruitment period. However, we argue that this situation would be replicated if we had collected our data from ED or the primary care setting.

Weights were collected using several sets of different scales available in the outpatients department. To promote standardisation of observed weights, it would have been preferable to use one set of scales; however, this does not reflect real clinical practice, where multiple sets of scales are used.

## Conclusions

Our study confirms that current UK ambulance weight guidance is inaccurate and may not reflect the weight of the wider paediatric population. Our findings question both the confidence with which a weight can be estimated based on age and the weights upon which current page for age guidance is based. All weight estimation formulae have limitations and result in a degree of imprecision. Our findings may not be applicable to all of the UK, and further opportunities exist to explore the impact that an accurate weight system can have in paramedic practice.

## Implications for practice

To our knowledge, this is the first study to evaluate the accuracy of paediatric weight guidance used in UK paramedic practice. Care derived from an accurate weight remains the gold standard and, when available, consideration should be given to using this rather than an age-based guide. Children are undoubtedly getting heavier, and the future challenge is to develop a formula that responds to this increase in weight and that accurately reflects the child population.

## Author contributions

KC designed the study, analysed and interpreted the data and wrote the article. MC and CM analysed and interpreted the data and provided critical revision of the article. KC acts as the guarantor for this article.

## Conflict of interest

None declared.

## Ethics

North East – Tyne & Wear South Research Ethics Committee (REC: 19/NE/0168) and the Health Research Authority have provided favourable ethical opinion and support for this research. As we used anonymised, routinely collected data, written informed consent was deemed unnecessary.

## Funding

None.
